# The relationship between physical activity and social anxiety in adolescents: a cross-lagged study

**DOI:** 10.3389/fpubh.2025.1752588

**Published:** 2026-01-12

**Authors:** Sihan Zhou, Wei Yang

**Affiliations:** 1 School of Law, Jiangsu University, Zhenjiang, China; 2 Department of Physical Education, Jiangsu University, Zhenjiang, China

**Keywords:** adolescents, cross-lagged panel model, longitudinal study, physical activity, social anxiety

## Abstract

**Background:**

Social anxiety (SA) is highly prevalent among adolescents and is closely linked to psychological wellbeing and social adjustment. Physical activity (PA) has been identified as an important protective factor; however, most existing studies rely on cross-sectional designs and focus on unidirectional effects, overlooking potential bidirectional dynamics between PA and SA.

**Objective:**

This study aimed to examine whether PA and SA exhibit stable bidirectional longitudinal associations in adolescents and to test their temporal stability and measurement invariance across three waves.

**Methods:**

A three-wave longitudinal study was conducted over 12 months with 989 adolescents (aged 10–14 years) from 3 junior middle schools in Jiangsu Province, China. PA was assessed using the PARS-3, and SA was measured using the Short Form Social Interaction Anxiety Scale and the Short Form Social Phobia Scale. Descriptive statistics and correlations were computed in SPSS 26.0. Measurement invariance and cross-lagged panel models (CLPMs) were estimated using Mplus 8.3.

**Results:**

(1) Both PA and SA demonstrated configural, metric, and scalar invariance across the three waves, supporting comparability over time. (2) PA and SA were significantly negatively correlated at all time points, and both variables showed substantial temporal stability. (3) CLPM results indicated that PA significantly negatively predicted subsequent SA (*β* = −0.259 to −0.273), while SA also significantly negatively predicted subsequent PA (*β* = −0.175 to −0.373), revealing a robust bidirectional negative longitudinal relationship.

**Conclusion:**

PA and SA influence each other dynamically over time: lower PA exacerbates SA, whereas higher SA reduces PA participation, forming a negative feedback loop. Future interventions hould simultaneously promote PA and reduce SA to break this maladaptive cycle and foster adolescents’ physical and psychological development.

## 1 Introduction

Social anxiety (SA) refers to a persistent and intense sense of fear and avoidance that individuals experience in social interaction contexts, typically manifested as excessive worry about negative evaluation, avoidance of social situations, and physiological discomfort symptoms (1). Originating from clinical psychology in the mid-20th century, the concept was later extended to adolescence as researchers observed that SA is also prominent during this developmental period (2). Wan et al. proposed that SA consists of three components: first, adolescents may feel tense and exhausted due to excessive social pressure; second, they may develop pessimistic emotions and feelings of detachment during interpersonal interactions; and third, they often experience a pervasive sense of social incompetence during adolescence (3). Existing research has shown that SA is highly prevalent among adolescents (4, 5). In China in particular, the combined influence of educational competition and digital social media means that adolescents are more likely to experience SA when facing increasingly complex interpersonal relationships and online pressures (6–8). Studying SA can not only improve adolescents’ social functioning (9, 10) but also reduce feelings and behaviors associated with social isolation (11, 12). Furthermore, as a multifaceted psychological state, SA negatively affects subjective wellbeing and mental health (13, 14). Studies have revealed that both external factors, such as school pressure and family events, and individual factors, including coping strategies and self-esteem, can influence adolescents’ levels of SA (15–17).

Physical activity (PA), an important research topic in health psychology in recent years, has been identified as a protective factor against SA (18–20). PA refers to planned exercise and daily movements that help maintain physical health, typically encompassing four structural dimensions: exercise intensity, frequency, duration, and type (21). The concept emerged from public health research in the late 20th century, and as scholars further explored the link between physical and mental health, they found that PA not only enhances physiological functioning but also regulates psychological states, which play an essential role in preventing and alleviating anxiety-related problems (22, 23). Empirical studies have demonstrated that higher levels of PA are generally associated with lower anxiety symptoms, greater social confidence, and better psychological regulation (24, 25). For example, regular physical exercise can promote the release of endorphins, enhance emotional stability, and strengthen individuals’ coping abilities in social situations (26, 27). In contrast, adolescents with insufficient PA are more likely to experience social withdrawal, reduced self-efficacy, and more severe mental health problems such as depression or avoidant personality tendencies (28, 29). Given that adolescents are highly sensitive to peer evaluation and social performance, those with elevated SA often avoid public or performance-oriented physical activities such as ball games or group-based dance because they worry about appearing incompetent or being negatively evaluated by peers. To reduce perceived social risks, they may gravitate toward solitary and low-visibility leisure activities, which further decrease their overall PA levels and may reinforce a cycle of avoidance and anxiety. Longitudinal evidence further shows that PA in adolescence is closely linked to social adjustment in adulthood, which highlights the importance of early intervention (30).

Although many scholars have quantitatively examined the relationship between PA and SA, most existing studies rely on cross-sectional data (31, 32), which makes it difficult to infer the directionality of this relationship. In addition, current analytical approaches often rely on assumptions derived from traditional regression models that conceptualize PA as exerting a one-way influence on SA (3). However, some studies suggest that SA may also reduce individuals’ engagement in PA (33, 34). Therefore, although previous research has revealed a correlation between PA and SA, many scholars have tended to conceptualize the relationship as one-directional without adequately considering its possible bidirectional nature.

**Figure 1:**
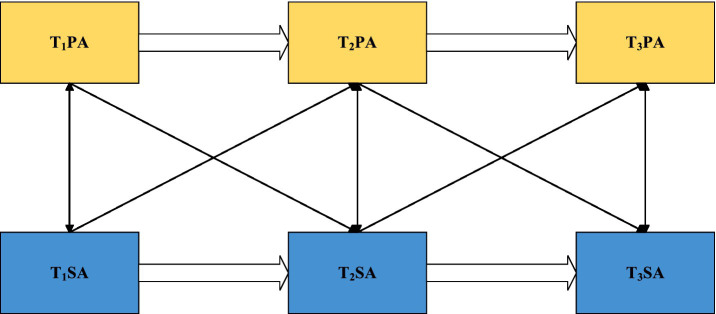
Cross-lagged model.

To address these limitations, the present study integrates Conservation of Resources Theory, Self-efficacy Theory, and the Health Behavior Model to explain the potential bidirectional dynamics between PA and SA. The Conservation of Resources Theory posits that physical activity functions as a resource-enhancing behavior that helps individuals buffer social stress and reduce anxiety (35). Self-efficacy theory further suggests that physical activity can enhance individuals’ perceived competence in social contexts (36), thereby alleviating social anxiety, whereas higher levels of social anxiety may undermine self-efficacy and reduce motivation to engage in physical activity. Meanwhile, the Health Behavior Model emphasizes that adolescents with elevated social anxiety are more likely to perceive exercise settings as socially threatening (37), which increases perceived barriers and leads to avoidance of physical activity. Integrating these theoretical perspectives, the present study proposes that PA and SA may reciprocally predict each other (see Figure 1). Therefore, a three-wave cross-lagged panel design was used to examine this bidirectional mechanism.

## 2 Materials and methods

### 2.1 Participants and procedure

This study adopted a three-wave longitudinal design over a 12-month period. Prior to data collection, *a priori* power analysis was conducted using G*Power 3.1 to determine the minimum sample size required for a repeated-measures ANOVA with within–between interactions. Input parameters included effect size *f* = 0.25, alpha = 0.05, power (1 − *β*) = 0.95, number of groups = 2, number of measurements = 3, correlation among repeated measures = 0, and non-sphericity correction *ε* = 0.5. According to Cohen (38), an effect size of *f* = 0.25 indicates a medium effect. The analysis suggested that at least 142 participants were required. We also referred to established guidelines for structural equation modeling, which recommend a sample size approximately 5 to 10 times the number of estimable parameters (39). Given that the present model included 15 free parameters, the required sample size should not be smaller than 150. Considering an anticipated attrition rate of approximately 20%, the theoretical minimum sample size was estimated to be 180 participants. To ensure sufficient statistical power and to account for possible participant attrition across the three waves, we recruited a considerably larger initial sample. In the first wave, a total of 1,137 questionnaires were collected. After excluding 39 invalid cases with more than 10% missing data or evident patterned responses, 1,098 valid responses remained.

In addition to meeting the minimum sample size requirements derived from the *a priori* power analysis and SEM guidelines, several practical and methodological considerations led us to recruit a substantially larger initial sample. First, the study involved three measurement waves across 1 year, and evidence from longitudinal research with adolescents indicates that attrition rates can be highly variable and may exceed 30% in school-based surveys. Therefore, oversampling was necessary to ensure an adequate final sample size after potential dropout. Second, data collection was conducted at the class level with the cooperation of entire schools, meaning that recruiting larger intact groups was more feasible and ethical than selectively reducing the number of participating students. Third, because cross-lagged panel models benefit from larger sample sizes for stable parameter estimation and increased statistical precision, recruiting a larger sample enhanced the reliability of the resulting estimates. Based on these practical and methodological considerations, we intentionally recruited far more participants than the theoretical minimum required.

Convenience sampling was used; however, to enhance the representativeness of the sample under practical constraints, a stratified selection approach was applied at the school level. Specifically, junior middle schools in Zhenjiang were first categorized according to geographical location (urban vs. rural) and school type (benchmark school vs. ordinary public school). Within each stratum, one school with accessible research conditions was selected, resulting in a final sample that included one urban key school, one urban ordinary school, and one rural ordinary school. This stratified convenience sampling ensured reasonable diversity in educational environments and socioeconomic backgrounds that may influence adolescents’ physical activity participation.

With school approval, group assessments were administered to students in the first year of middle school. To minimize potential interference from holidays, all three waves of data collection took place during weekday self-study periods and were intentionally scheduled away from major Chinese public holidays. Measurement times were as follows: Time 1 (T1), 15–19 April 2024; Time 2 (T2), 13–16 October 2024; and Time 3 (T3), 14–18 April 2025. All sessions were administered by trained research assistants in classroom settings, and students were instructed to complete the questionnaires independently in a quiet and distraction-free environment. Written informed consent was obtained from both students and their parents, and all participants were informed that participation was voluntary and anonymous and that withdrawal was permitted at any stage. To enable matching across waves, each participant was assigned a unique identification code, and data screening was conducted after each wave based on missing data rates and response quality.

In the second wave, 1,103 questionnaires were collected, of which 1,065 were valid. In the third wave, 1,059 questionnaires were collected, with 999 valid responses. Ultimately, 989 students completed all three waves and passed all quality checks, forming the final longitudinal sample for analysis. This sample consisted of 443 boys (44.8%) and 546 girls (55.2%), with 475 urban students (48.0%) and 514 rural students (52.0%). At T1, participants were between 10 and 14 years old (M = 12.39, SD = 0.68).

To evaluate the presence of systematic attrition, we created a variable labeled “data completeness” (0 = complete data across all three waves, 1 = missing data) and compared the complete-data group with the missing-data group on key demographic and study variables. Based on the 1,098 valid T1 cases, 989 were classified as the complete-data group and 109 as the missing-data group. Chi-square tests indicated no significant differences between groups in gender distribution (χ^2^ = 3.02, *p* = 0.08) or urban–rural background (χ^2^ = 1.47, *p* = 0.23). Independent-samples t-tests were then conducted using T1 data, and the results showed no significant differences between the two groups in T1 PA (t = −0.84, *p* = 0.40) and T1 SA (t = 1.09, *p* = 0.28). These findings indicate that attrition in this study was not systematically associated with gender, urban–rural background, or the key study variables (PA and SA).

### 2.2 Measures

#### 2.2.1 Physical activity

PA was assessed using the Physical Activity Rating Scale (PARS-3) developed by Liang (40). The scale evaluates an individual’s PA level across three dimensions: intensity, duration, and frequency. Intensity is rated on a 5-point scale based on participants’ subjective perception, ranging from 1 (“light activity”) to 5 (“vigorous and long-lasting activity accompanied by rapid breathing and heavy sweating”). Frequency is rated from 1 (“less than once per month”) to 5 (“once every day”). Duration is rated from 0 (“less than 10 min”) to 4 (“more than 60 min”). The total PA score is calculated by multiplying the scores of intensity, duration, and frequency, yielding a possible range of 0 to 100. In the present sample, the scale demonstrated good internal consistency, with Cronbach’s alpha coefficients of 0.773 (T1), 0.748 (T2), and 0.715 (T3).

#### 2.2.2 Social anxiety

SA was measured using the Short Form Social Interaction Anxiety Scale and the Short Form Social Phobia Scale, developed by Peters et al. (41). Each short form contains 6 items, resulting in a total of 12 items. All items are rated on a 5-point Likert scale ranging from 0 (“strongly disagree”) to 4 (“strongly agree”). SA total scores are obtained by summing all item scores, with higher scores indicating higher levels of SA. This combined measure has been widely used and validated in Chinese populations (42, 43). In the present sample, internal consistency was high, with Cronbach’s alpha coefficients of 0.884 (T1), 0.878 (T2), and 0.853 (T3).

### 2.3 Data analysis

Data analysis was conducted in two stages. First, all valid questionnaires from the three waves were entered into SPSS 26.0, and descriptive statistics were calculated for all variables at each time point. Second, before modeling the longitudinal associations, longitudinal measurement invariance of the SA scale across the three waves was examined using Mplus 8.3. Three levels of invariance were tested sequentially: configural invariance (identical factor structure), metric invariance (equal factor loadings), and scalar invariance (equal item intercepts). Model fit was evaluated using chi-square (χ^2^), degrees of freedom (df), comparative fit index (CFI), Tucker–Lewis index (TLI), standardized root mean square residual (SRMR), and root mean square error of approximation (RMSEA). Measurement invariance was considered acceptable when changes in fit indices between nested models met established criteria (ΔCFI ≤ 0.01, ΔTLI ≤ 0.01) (44). To examine the longitudinal associations between PA and SA, a three-wave cross-lagged panel model (CLPM) was constructed and estimated in Mplus 8.3. The model included autoregressive effects, which capture the stability of each construct over time, as well as cross-lagged effects, which assess the extent to which one construct predicts the other at subsequent time points.

## 3 Results

### 3.1 Common method bias test

Harman’s single-factor test was used to examine common method bias. Exploratory factor analyses of the T1, T2, and T3 data extracted three factors with eigenvalues greater than 1 in each wave. The variance explained by the first factors was 27.647, 27.162, and 34.046%, respectively, all of which are below the 40% threshold (45). These results indicate that common method bias was not a serious concern in this study.

### 3.2 Measurement invariance analysis

Before testing the longitudinal relationships among the main variables, it was necessary to establish the measurement stability of the PA scale and the SA scale across the three waves. Using nested model comparisons, we sequentially tested configural, metric, and scalar invariance.

As shown in Table 1, the baseline configural models for both constructs demonstrated excellent fit indices, supporting the stability of their factor structures over time. Next, equality constraints were added stepwise to test higher levels of invariance. When factor loadings and intercepts were constrained to be equal across time points, changes in model fit (ΔCFI and ΔTLI) remained below the cutoff of 0.01, indicating that model fit did not deteriorate significantly.

**Table 1:** Testing for longitudinal invariance of data from three surveys.

Variable	Model	χ^2^/df	CFI	TLI	SRMR	RMSEA	Model comparison	ΔCFI	ΔTLI
PA	M1	0	1	1	0	0			
	M2	1.805	0.998	0.996	0.020	0.029	M2-M1	−0.002	−0.004
	M3	1.952	0.996	0.996	0.019	0.031	M3-M2	−0.002	0
SA	M1	1.842	0.991	0.989	0.019	0.029			
	M2	1.690	0.992	0.991	0.020	0.026	M2-M1	0.001	0.002
	M3	1.664	0.992	0.992	0.021	0.026	M3-M2	0	0.001

M1 = configural model; M2 = metric model; M3 = scalar model; PA = physical activity; SA = social anxiety.

### 3.3 Descriptive statistics

Table 2 presents descriptive statistics and Pearson correlations for all variables. Tests of normality showed that the absolute values of skewness and kurtosis for all variables were well below the critical thresholds, indicating that the data met the assumptions of approximate normality. Correlation analyses revealed that PA and SA were significantly negatively correlated at all three time points. In addition, both variables showed significant moderate to strong positive correlations across T1, T2, and T3, confirming the relative stability of PA and SA over time and providing support for subsequent tests of longitudinal cross-lagged effects.

**Table 2:** Correlations and descriptive statistics for PA and SA across three time points.

Variable	T1PA	T2PA	T3PA	T1SA	T2SA	T3SA
T1PA	1					
T2PA	0.468**	1				
T3PA	0.398**	0.512**	1			
T1SA	−0.373**	−0.398**	−0.375**	1		
T2SA	−0.436**	−0.456**	−0.450**	0.532**	1	
T3SA	−0.358**	−0.438**	−0.429**	0.497**	0.513**	1
Min	0	0	0	0.17	0	0.08
Max	100	100	100	4.33	4.25	3.92
Mean	31.02	33.79	34.20	1.96	1.83	1.94
SD	30.08	31.95	29.94	0.98	0.94	0.84
Skewness	0.719	0.584	0.539	0.328	0.492	−0.004
Kurtosis	−0.707	−1.078	−0.926	−0.411	−0.202	−0.473

**p < 0.01.

### 3.4 Cross-lagged analysis

Based on the correlation analyses, a series of cross-lagged models was constructed in Mplus 8.3 to examine the longitudinal relationships between PA and SA. Four competing models were specified: M1 was an autoregressive model that included only the stability paths of each variable across time. M2 added unidirectional paths from PA at an earlier time point to SA at the subsequent time point, in addition to the autoregressive paths. M3 added unidirectional paths from SA at an earlier time point to PA at the subsequent time point, in addition to the autoregressive paths. M4 was a full cross-lagged model that included all autoregressive paths and all bidirectional predictive paths between PA and SA. As shown in Table 3, the key fit indices of M1, M2, and M3 did not meet acceptable statistical standards. In contrast, M4 demonstrated the best and acceptable overall fit (χ^2^ = 16.729, df = 4, CFI = 0.944, TLI = 0.905, SRMR = 0.045, RMSEA = 0.053), indicating that this model most accurately captured the longitudinal associations between the variables. Therefore, M4 was selected as the final model for subsequent analyses.

**Table 3:** Model fit indices for the four cross-lagged panel models.

Path	χ^2^	df	CFI	TLI	SRMR	RMSEA
M1	487.9	9	0.713	0.553	0.201	0.232
M2	307.821	7	0.82	0.639	0.143	0.208
M3	317.282	7	0.814	0.628	0.136	0.212
M4	16.729	4	0.944	0.905	0.045	0.053

**Figure 2:**
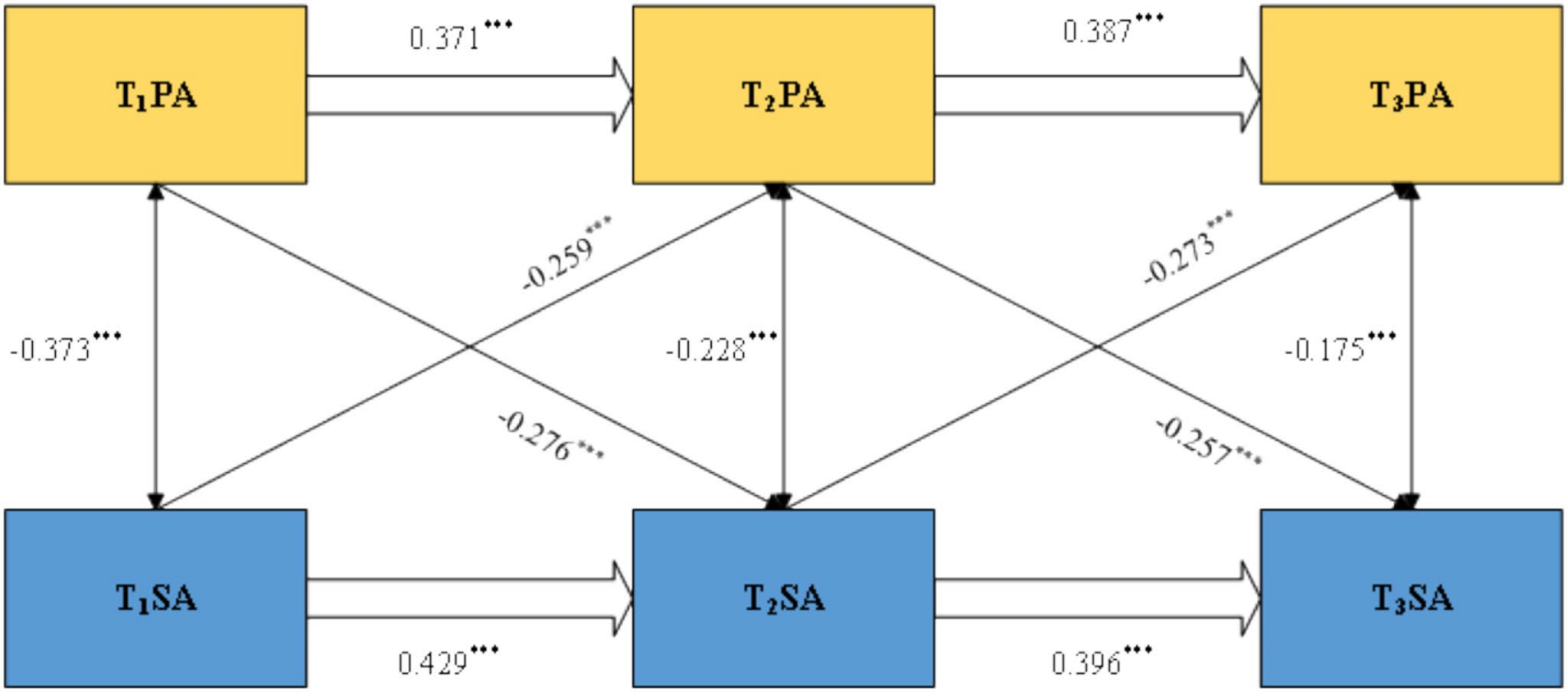
Standardized path coefficients of the three-wave cross-lagged model between PA and SA. ***p < 0.001.

Figure 2 presents the standardized path coefficients of Model M4. First, regarding the autoregressive effects, PA at T1 significantly and positively predicted PA at T2 (*β* = 0.371, *p* < 0.001), and PA at T2 predicted PA at T3 (*β* = 0.387, *p* < 0.001). Similarly, SA at T1 significantly and positively predicted SA at T2 (*β* = 0.429, *p* < 0.001), and SA at T2 predicted SA at T3 (*β* = 0.396, *p* < 0.001), indicating high temporal stability for both PA and SA.

Second, regarding the cross-lagged effects, PA at T1 significantly and negatively predicted SA at T2 (*β* = −0.259, *p* < 0.001), and PA at T2 predicted SA at T3 (*β* = −0.273, *p* < 0.001). The reverse paths were also significant: SA at T1 significantly and negatively predicted PA at T2 (*β* = −0.373, *p* < 0.001), and SA at T2 significantly and negatively predicted PA at T3 (*β* = −0.175, *p* < 0.001). These findings suggest that PA and SA have a significant negative correlation over time.

## 4 Discussion

The descriptive statistics in this study showed that adolescents’ PA scores remained consistent across all three measurement waves, aligning with findings from previous studies (46, 47) and suggesting relatively stable PA levels among adolescents. This may be because adolescents are in a phase of rapid physical development, during which exercise habits are more easily formed, and participation tends to be more consistent. In contrast, adolescents’ SA scores across the three waves were higher than those reported in studies conducted in other countries (5, 8), a difference that may stem from cultural characteristics. Specifically, Chinese adolescents tend to emphasize collective activities and family interactions, cultural elements that may exacerbate the negative impact of social pressure (48, 49). For example, in a collectivistic cultural context, Chinese adolescents often face higher social expectations and concerns related to “losing face” or disappointing their families, which may heighten fear of negative evaluation and result in stronger avoidance behaviors and physiological discomfort (50). Moreover, compared with Western individualistic cultures, Chinese adolescents experience heightened social competition due to an education system that emphasizes high-stakes achievement (such as the college entrance examination) and digital media environments that encourage performance comparison and online exposure (51, 52). These factors may increase emotional distress and undermine social confidence and coping abilities (53).

The findings also showed that PA negatively predicted SA, consistent with previous research (3, 54). According to the Conservation of Resources theory (35), individuals experience anxiety symptoms when their resources are depleted (55). PA represents a personal resource; low levels of PA may be perceived as a loss of resources, thereby eliciting SA (27, 56). Chen et al. (57) found that individuals with higher PA tend to adopt effective psychological regulation strategies. When encountering persistent negative social situations, they engage in greater physical activity and bodily relaxation as a form of self-protection, which prevents emotional overload and reduces perceived stress, thereby indirectly lowering SA (22, 58). Wu et al. (59) further demonstrated that PA not only directly predicts lower SA but also promotes more active coping strategies, helping individuals better utilize their physical and psychological resources when facing social challenges, which in turn reduces SA behaviors.

This study also found that SA negatively predicted PA. Longitudinal findings in other populations suggest that certain components of SA can predict future patterns of PA (31, 33). According to the Health Behavior Model (37), individuals’ behavioral patterns are shaped by their perceptions of health-related benefits, barriers, and self-regulatory abilities, all of which influence how they engage in behaviors such as physical activity and how they experience psychological outcomes. Higher levels of SA are typically accompanied by negative social behaviors, and prolonged negative interaction patterns can weaken individuals’ perceptions of their own ability and value (60, 61). This reduces motivation and persistence for PA. In summary, SA leads individuals to form negative interpersonal perceptions during social interactions, which diminishes their interest and engagement in PA. Additionally, the negative social behaviors associated with high SA can further hinder the development of PA.

The cross-lagged model revealed a bidirectional predictive relationship between PA and SA, indicating a complex multidimensional interaction. One longitudinal study found reciprocal predictive effects between self-esteem and anxiety, suggesting interactive components between emotional and behavioral traits (62). Additionally, a meta-analysis showed that self-efficacy significantly contributes to avoidance behaviors in anxiety, while reductions in anxiety enhance self-efficacy (63), further supporting bidirectional interaction. Self-efficacy theory provides a useful explanation for this reciprocal relation: individuals’ beliefs about their ability to successfully perform a behavior shape their emotional reactions, motivation, and behavioral choices. Higher self-efficacy promotes greater engagement in physical activity and more adaptive emotional regulation, whereas lower self-efficacy increases vulnerability to negative emotional states (36). Components of PA interact dynamically within social contexts, shaping emotional states, and behavioral choices (64). Conversely, SA, as a complex emotional and behavioral state, significantly influences PA patterns. Within this framework, an individual’s bodily regulation in social contexts shapes the severity of anxiety, while chronic anxiety can undermine the stability and trajectory of PA (65, 66). Together, PA and SA form a feedback mechanism, highlighting the complexity of emotional and behavioral responses to social challenges.

A notable observation in the cross-lagged model was that within-time correlations were higher than cross-time autocorrelations. This is likely because variables measured at the same time point are influenced by shared situational factors, whereas associations across time are weakened by temporal fluctuations and random disturbances. Bühler et al. (67) noted that long-term rank-order stability in personality declines sharply as time lags increase. Dapp et al. similarly argued (68) that autocorrelations weaken over longer intervals, reflecting natural changes in personality variables. Given the extended intervals in this study, both PA and SA were exposed to external influences, resulting in lower cross-time stability relative to within-time associations. This suggests that the bidirectional relationship between PA and SA is shaped by both internal individual factors and external environmental conditions. For instance, physical self-efficacy can influence SA through social support (69), and SA trajectories can be moderated by resilience and self-efficacy (70). These findings suggest the presence of additional latent variables not captured in the current model, which future research should investigate.

### 4.1 Implications and limitations

Overall, this study expands theoretical perspectives on the relationship between PA and SA and offers practical insight for adolescent mental health. Unlike prior research, our findings suggest that SA is not merely influenced by PA but may also negatively predict PA, forming a bidirectional causal relationship. This contributes to the theoretical understanding of PA and provides a new perspective on the long-term consequences of SA. Based on these findings, future interventions should simultaneously aim to enhance PA and reduce SA. Prevention-oriented strategies should prioritize alleviating SA to prevent escalation into more severe mental-health problems. Educators and practitioners should adopt dual-pathway interventions that strengthen PA habits and improve social experiences and emotional regulation. Considering environmental factors such as social support will help create a positive feedback loop that promotes adolescents’ self-development, social functioning, and psychological wellbeing.

Despite its contributions, this study has several limitations that should be acknowledged. First, PA was measured solely through self-report using the PARS-3, which may be affected by recall bias and social desirability, especially when adolescents are asked to estimate exercise frequency and intensity over relatively long periods. Future studies should consider combining subjective ratings with objective indicators (for example, accelerometers, wearable trackers, or smartphone-based step counts and heart-rate data) to more accurately capture different dimensions of PA, including intensity, duration, and type. Second, although we identified robust associations between PA and SA, the present study did not differentiate specific forms or contexts of PA. It is plausible that team sports or cooperative activities (for example, basketball and football) may have stronger effects on social confidence and interpersonal skills than solitary activities such as jogging or swimming, whereas highly competitive sports might even exacerbate performance-related anxiety in some adolescents. Distinguishing between organized versus informal activities, competitive versus recreational sports, and indoor versus outdoor contexts would allow a more fine-grained understanding of which types of PA are particularly beneficial for adolescents with elevated SA.

Third, while the three-wave longitudinal design with approximately 6-month intervals strengthen temporal inference compared with cross-sectional designs, the fixed and relatively long-time lags may not fully capture the short-term dynamics through which PA and SA influence each other. Reciprocal effects may also unfold on micro-timescales (for example, daily or weekly fluctuations) or, conversely, require longer follow-up periods to stabilize. Future research could adopt intensive longitudinal methods such as ecological momentary assessment (EMA), daily diary approaches, or weekly follow-ups to examine how within-person changes in PA and affect co-occur in real time and whether the bidirectional associations observed here also operate on shorter time scales. Fourth, although we controlled for several basic demographic characteristics, unmeasured confounders may still have influenced the observed relationships. For instance, body mass index (BMI), body image dissatisfaction, and weight-related teasing may shape both adolescents’ willingness to engage in public exercise and their levels of SA. Similarly, personality traits such as introversion, neuroticism, or behavioral inhibition, as well as family functioning and parenting style, may predispose individuals to both lower PA and higher SA.

Finally, the sample characteristics limit the generalizability of the findings. Participants were drawn from three junior middle schools in a single province, and all were typically developing adolescents without reported physical disabilities or severe mobility constraints. As a result, the present conclusions may not extend to adolescents from other regions or educational stages, nor to youth with chronic illnesses, physical impairments, or special educational needs, whose opportunities and motivations for PA, as well as their experiences of SA, may differ substantially.

To address these limitations, future studies should incorporate a richer set of covariates (for example, BMI, body image indices, personality traits, and family functioning) to test the robustness of the bidirectional PA–SA model more rigorously and to reduce the risk of omitted-variable bias. In addition, expanding the model to include empirically measured mediators such as self-esteem, physical self-efficacy, perceived social support, emotion regulation strategies, or physiological stress markers (for example, heart-rate variability, cortisol) would help clarify the mechanisms through which PA and SA influence each other over time. Multimethod and multi-informant approaches, combining self-report, objective PA monitoring, EMA, and possibly teacher or parent reports, could further improve measurement precision and ecological validity, and provide a more comprehensive picture of how physical and social–emotional development are intertwined in adolescence.

## 5 Conclusion

Through a three-wave longitudinal investigation, this study demonstrated a stable bidirectional negative predictive relationship between PA and SA among adolescents. Low PA was found to exacerbate SA, while high levels of SA reduced PA participation, forming a detrimental cycle. These findings highlight the need for integrated interventions. Future health promotion strategies should break the mind–body divide by combining regular PA with psychological regulation, thereby interrupting the negative feedback loop and supporting the holistic development of adolescents’ physical and mental wellbeing.

## Data Availability

The original contributions presented in the study are included in the article/supplementary material, further inquiries can be directed to the corresponding author.

## References

[1] Hofmann SG. Fear of positive evaluation and the bivalent fear of evaluation model of social anxiety: an integration. J Anxiety Disord. (2025) 111:102986. doi: 10.1016/j.janxdis.2025.102986, 39951864

[2] Chiu K Clark DM Leigh E. Prospective associations between peer functioning and social anxiety in adolescents: a systematic review and meta-analysis. J Affect Disord. (2021) 279:650–61. doi: 10.1016/j.jad.2020.10.055, 33190116 PMC7758784

[3] Wan H Huang W Zhang W Hu C. Exploring adolescents’ social anxiety, physical activity, and Core self-evaluation: a latent profile and mediation approach. Int J Ment Health Promot. (2025) 27:1611–26. doi: 10.32604/ijmhp.2025.070457, 40612875

[4] Rapee RM Oar EL Johnco CJ Forbes MK Fardouly J Magson NR . Adolescent development and risk for the onset of social-emotional disorders: a review and conceptual model. Behav Res Ther. (2019) 123:103501. doi: 10.1016/j.brat.2019.103501, 31733812

[5] Jefferies P Ungar M. Social anxiety in young people: a prevalence study in seven countries. PLoS One. (2020) 15:e0239133. doi: 10.1371/journal.pone.0239133, 32941482 PMC7498107

[6] Ding N Xu Z. China adolescents comparisons on social media and emotional eating: a moderated analysis. Child Adolesc Soc Work J. (2021) 40:107–17. doi: 10.1007/s10560-021-00750-3, 41415797

[7] Zhu X Lian W Fan L. Network analysis of internet addiction, online social anxiety, fear of missing out, and interpersonal sensitivity among Chinese university students. Depress Anxiety. (2024) 2024:1–14. doi: 10.1155/2024/5447802, 40226693 PMC11918617

[8] Xin S Peng H Sheng L. Changes of social anxiety in Chinese adolescents during 2002∼2020: an increasing trend and its relationship with social change. Child Youth Serv Rev. (2022) 142:106614. doi: 10.1016/j.childyouth.2022.106614

[9] Xian J Zhang Y Jiang B. Psychological interventions for social anxiety disorder in children and adolescents: a systematic review and network meta-analysis. J Affect Disord. (2024) 365:614–27. doi: 10.1016/j.jad.2024.08.097, 39173929

[10] Figueiredo DV Alves F Vagos P. Psychological inflexibility explains social anxiety over time: a mediation analyses with a clinical adolescent sample. Curr Psychol. (2024) 43:4404–15. doi: 10.1007/s12144-023-04650-w, 37359612 PMC10117271

[11] Sackl-Pammer P Jahn R Özlü-Erkilic Z Pollak E Ohmann S Schwarzenberg J . Social anxiety disorder and emotion regulation problems in adolescents. Child Adolesc Psychiatry Ment Health. (2019) 13:37. doi: 10.1186/s13034-019-0297-9, 31583014 PMC6771087

[12] Walder N Berger T Schmidt SJ. Prevention and treatment of social anxiety disorder in adolescents: protocol for a randomized controlled trial of the online guided self-help intervention SOPHIE. JMIR Res Protoc. (2023) 12:e44346. doi: 10.2196/44346, 37342086 PMC10337443

[13] Ye B Li L Wang P Wang R Liu M Wang X . Social anxiety and subjective well-being among Chinese college students: a moderated mediation model. Pers Individ Differ. (2021) 175:110680. doi: 10.1016/j.paid.2021.110680

[14] Lee Y Jeon YJ Kang S Shin JI Jung Y-C Jung SJ. Social media use and mental health during the COVID-19 pandemic in young adults: a meta-analysis of 14 cross-sectional studies. BMC Public Health. (2022) 22:995. doi: 10.1186/s12889-022-13409-0, 35581597 PMC9112239

[15] Antonietti C Camerini A-L Marciano L. The impact of self-esteem, family and peer cohesion on social appearance anxiety in adolescence: examination of the mediating role of coping. Int J Adolesc Youth. (2020) 25:1089–102. doi: 10.1080/02673843.2020.1858888

[16] Gao M Liu W. Exploring family functioning and adolescent academic anxiety: emotional stability and social support as mediators. Psychol Res Behav Manag. (2025) 18:1111–24. doi: 10.2147/prbm.s508537, 40406340 PMC12094906

[17] Wang Y Yan X Liu L Lu X Luo L Ding X. The influence of vulnerable narcissism on social anxiety among adolescents: the mediating role of self-concept clarity and self-esteem. Int J Ment Health Promot. (2024) 26:429–38. doi: 10.32604/ijmhp.2024.050445

[18] Chen X Huang W Hu C. Associations of perceived teacher–student relationship and friendship quality with adolescents’ interest in physical education: a latent profile analysis. Front Sports Act Living. (2025) 7:1677083. doi: 10.3389/fspor.2025.1677083, 41323867 PMC12657499

[19] Yuan Y Huang W Hu C Zhang W. The interaction of physical activity and sleep quality with depression and anxiety in older adults. Front Public Health. (2025) 13:1674459. doi: 10.3389/fpubh.2025.1674459, 41211411 PMC12588915

[20] Hu C Zhang W Huang W. The role of self-objectification and physical exercise in social appearance anxiety and restrained eating among female college students. Behav Sci. (2025) 15:1300. doi: 10.3390/bs15101300, 41153090 PMC12561914

[21] Hu C Zhang W Huang W Jin C. How grit enhances physical exercise in college students: mediating roles of personal growth initiative and self-efficacy. Front Psychol. (2025) 16:1652984. doi: 10.3389/fpsyg.2025.1652984, 40994858 PMC12455857

[22] Huang W Chen B Hu C. The latent profile structure of negative emotion in female college students and its impact on eating behavior: the mediating role of physical exercise. Front Public Health. (2025) 13:1663474. doi: 10.3389/fpubh.2025.1663474, 40880924 PMC12380551

[23] Hu C. Letter to the editor regarding: “leisure-time physical activity patterns and predictors in patients before and after metabolic and bariatric surgery: a cross-sectional study.”. Obes Surg. (2025). doi: 10.1007/s11695-025-08248-y 40921960

[24] Wang T Nie Y Yao X Zhang J Li Y Sun H . The chain mediating role of emotion regulation and stress perception in physical activity alleviating college students’ health anxiety. Sci Rep. (2025) 15:29189. doi: 10.1038/s41598-025-14481-3, 40783609 PMC12335499

[25] Singh B Olds T Curtis R Dumuid D Virgara R Watson A . Effectiveness of physical activity interventions for improving depression, anxiety and distress: an overview of systematic reviews. Br J Sports Med. (2023) 57:1203–9. doi: 10.1136/bjsports-2022-106195 36796860 PMC10579187

[26] Huang W Chen B Hu C. Exploring self-rated health, physical activity, and social anxiety among female Chinese university students: a variable- and person-centered analysis. Front Public Health. (2025) 13:1681504. doi: 10.3389/fpubh.2025.1681504, 41048258 PMC12488654

[27] Chen B Huang W Hu C. The relationship between positive exercise experiences and mobile phone addiction tendencies in older adults: a cross-lagged study. Front Public Health. (2025) 13:1710048. doi: 10.3389/fpubh.2025.1710048, 41323583 PMC12661996

[28] Lin H Chen H Liu Q Xu J Li S. A meta-analysis of the relationship between social support and physical activity in adolescents: the mediating role of self-efficacy. Front Psychol. (2024) 14:1305425. doi: 10.3389/fpsyg.2023.1305425, 38282843 PMC10811609

[29] Rutter LA Thompson HM Howard J Riley TN De Jesús-Romero R Lorenzo-Luaces L. Social media use, physical activity, and internalizing symptoms in adolescence: cross-sectional analysis. JMIR Ment Health. (2020) 8:e26134. doi: 10.2196/preprints.26134 PMC8482183 34524096

[30] Doré I O’Loughlin JL Schnitzer ME Datta GD Fournier L. The longitudinal association between the context of physical activity and mental health in early adulthood. Ment Health Phys Act. (2018) 14:121–30. doi: 10.1016/j.mhpa.2018.04.001

[31] Nie Y Wang W Liu C Wang T Zhou F Gao J. Social challenges on university campuses: how does physical activity affect social anxiety? The dual roles of loneliness and gender. Behav Sci. (2025) 15:1063. doi: 10.3390/bs15081063, 40867420 PMC12382821

[32] Liu Y Nie Z Dong H Shou L Xu J Cao Q . The mediating role of sleep quality on the relationship between physical activity and social anxiety disorder among Chinese college freshmen. Front Psychol. (2025) 16:1599041. doi: 10.3389/fpsyg.2025.1599041, 40735178 PMC12305814

[33] Zartaloudi A Christopoulos D. Social physique anxiety and physical activity. Eur Psychiatry. (2021) 64:S759. doi: 10.1192/j.eurpsy.2021.2011

[34] Kowalska J. The level of stress and anxiety in pregnant women depending on social support and physical activity. J Clin Med. (2023) 12:3143. doi: 10.3390/jcm12093143, 37176585 PMC10179597

[35] Ng T. Conservation of resources theory In: Wiley Encyclopedia of management. Chichester, UK: Wiley‑Blackwell. (2015). 1–1.

[36] Bandura A. Self-efficacy mechanism in human agency. Am Psychol. (1982) 37:122–47. doi: 10.1037/0003-066X.37.2.122

[37] Carpenter CJ. A meta-analysis of the effectiveness of health belief model variables in predicting behavior. Health Commun. (2010) 25:661–9. doi: 10.1080/10410236.2010.521906, 21153982

[38] Cohen J. Quantitative methods in psychology a power primer. (1992). Available online at: https://consensus.app/papers/quantitative-methods-in-psychology-a-power-primer-cohen/a054ea444aa555fb8a81a0ca4b650d1d/ (Accessed November 22, 2025)

[39] Wolf EJ Harrington KM Clark SL Miller MW. Sample size requirements for structural equation models. Educ Psychol Meas. (2013) 73:913–34. doi: 10.1177/0013164413495237, 25705052 PMC4334479

[40] Liang D. Stress levels among college students and their relationship with physical exercise. Chin Ment Health J. (1994) 8:5–6.

[41] Peters L Sunderland M Andrews G Rapee RM Mattick RP. Development of a short form social interaction anxiety (SIAS) and social phobia scale (SPS) using nonparametric item response theory: the SIAS-6 and the SPS-6. Psychol Assessment. (2012) 24:66–76. doi: 10.1037/a0024544, 21744971

[42] Li X Liu Y Rong F Wang R Li L Wei R . Physical activity and social anxiety symptoms among Chinese college students: a serial mediation model of psychological resilience and sleep problems. BMC Psychol. (2024) 12:440. doi: 10.1186/s40359-024-01937-w, 39138553 PMC11323702

[43] Chau AKC So SH Sun X Zhu C Chiu C-D Chan RCK . The co-occurrence of multidimensional loneliness with depression, social anxiety and paranoia in non-clinical young adults: a latent profile analysis. Front Psych. (2022) 13:931558. doi: 10.3389/fpsyt.2022.931558, 36186883 PMC9517946

[44] Putnick DL Bornstein MH. Measurement invariance conventions and reporting: the state of the art and future directions for psychological research. Dev Rev. (2016) 41:71–90. doi: 10.1016/j.dr.2016.06.004, 27942093 PMC5145197

[45] Podsakoff PM MacKenzie SB Lee J-Y Podsakoff NP. Common method biases in behavioral research: a critical review of the literature and recommended remedies. J Appl Psychol. (2003) 88:879–903. doi: 10.1037/0021-9010.88.5.879, 14516251

[46] Yuan Y Hu W Hu C Zhang W Song C. Mediation and latent variable analysis of new curriculum standard-based physical education core literacy and subjective exercise experience among middle school students. Chin J Sch Health. (2025) 46:941–5. doi: 10.16835/j.cnki.1000-9817.2025205

[47] Bertrand C Steinberg L Duell N Di Giunta L Dodge KA Gurdal S . Physical activity and two-year change in adolescent well-being in nine countries. J Res Adolesc. (2025) 35:e70035. doi: 10.1111/jora.70035, 40411243 PMC12232549

[48] Zhang C Zhang Q Zhuang H Xu W. The reciprocal relationship between depression, social anxiety and aggression in Chinese adolescents: the moderating effects of family functioning. J Affect Disord. (2023) 329:379–84. doi: 10.1016/j.jad.2023.02.134, 36870452

[49] Wang J Huang X Li Z Chen K Jin Z He J . Effect of parenting style on the emotional and behavioral problems among Chinese adolescents: the mediating effect of resilience. BMC Public Health. (2024) 24:787. doi: 10.1186/s12889-024-18167-9, 38481184 PMC10935827

[50] Dang H-M Le T Chau C Nguyen PT Weiss B. Individualism and collectivism as moderators of relations between adverse childhood experiences and adolescent aggressive behavior. Res Child Adolesc Psychopathol. (2025) 53:569–81. doi: 10.1007/s10802-025-01296-z, 40014281 PMC12031781

[51] Ye L Posada A Liu Y. A review on the relationship between Chinese adolescents’ stress and academic achievement. New Dir Child Adolesc Dev. (2019) 2019:81–95. doi: 10.1002/cad.20265, 30614631

[52] Li J Zhang N Yao M Xing H Liu H. Academic social comparison and depression in Chinese adolescents: the mediating role of basic psychological needs satisfaction. Sch Ment Health. (2021) 13:719–29. doi: 10.1007/s12310-021-09436-8

[53] Zhang W Huang W Hu C Yuan Y Chen X. The impact of physical activity and dietary behavior on depression in college students: a study on mediation effects and network analysis. Front Public Health. (2025) 13:1683468. doi: 10.3389/fpubh.2025.1683468, 41132184 PMC12540118

[54] Zhao H Chi Y Shi L. Analyzing the effect of physical exercise on social anxiety in college students using the chain mediation model. Sci Rep. (2025) 15:17751. doi: 10.1038/s41598-025-02445-6, 40404703 PMC12098904

[55] Hobfoll SE. Conservation of resources theory: Its implication for stress, health, and resilience. Oxford: Oxford University Press. (2010)

[56] Yuan Y Yang J Huang W Hu C Zhang W Chen B. Relationships among anxiety, psychological resilience, and physical activity in university students: variable-centred and person-centred perspectives. Front Psychol. (2025) 16:1694344. doi: 10.3389/fpsyg.2025.1694344, 41356033 PMC12679388

[57] Chen B Huang W Zhang W Yang C Hu C. The latent profile structure of alexithymia in the elderly and its relationship to eating behaviors: the mediating role of physical activity. Front Psychol. (2025) 16:1677083. doi: 10.3389/fpsyg.2025.1701168, 41383413 PMC12690933

[58] Yang D Hu C Zhou Z He L Huang S Wan M . The impact of perceived stigma on appearance anxiety in postoperative rhinoplasty patients: a variable-centered and person-centered perspective. Acta Psychol. (2025) 260:105660. doi: 10.1016/j.actpsy.2025.105660, 41086734

[59] Wu J Shao Y Hu J Zhao X. The impact of physical exercise on adolescent social anxiety: the serial mediating effects of sports self-efficacy and expressive suppression. BMC Sports Sci Med Rehabil. (2025) 17:57. doi: 10.1186/s13102-025-01107-4, 40121514 PMC11929206

[60] Micheal Raj APP Babu S. Social anxiety among college students and its relation to negative self-portrayal. Mind Soc. (2022) 11:41–6. doi: 10.56011/mind-mri-111-20225

[61] Hume Figueroa MR Téllez Alanís MDLCB. Self-esteem and self-concept as predictors of social anxiety in university students. Rev Educ. (2025) 49:1–20. doi: 10.15517/revedu.v49i1.61007, 41417743

[62] Li W Guo Y Lai W Wang W Li X Zhu L . Reciprocal relationships between self-esteem, coping styles and anxiety symptoms among adolescents: between-person and within-person effects. Child Adolesc Psychiatry Ment Health. (2023) 17:21. doi: 10.1186/s13034-023-00564-4, 36755330 PMC9909938

[63] Lau SC Bhattacharjya S Fong MW Nicol GE Lenze EJ Baum C . Effectiveness of theory-based digital self-management interventions for improving depression, anxiety, fatigue and self-efficacy in people with neurological disorders: a systematic review and meta-analysis. J Telemed Telecare. (2022) 28:547–58. doi: 10.1177/1357633x20955122, 32954920 PMC8145956

[64] Wang C. The role of physical activity promoting thinking skills and emotional behavior of preschool children. Psicol Reflex Crit. (2022) 35:24. doi: 10.1186/s41155-022-00223-1, 35913559 PMC9343512

[65] Kandola A Vancampfort D Herring M Rebar A Hallgren M Firth J . Moving to beat anxiety: epidemiology and therapeutic issues with physical activity for anxiety. Curr Psychiatry Rep. (2018) 20:63. doi: 10.1007/s11920-018-0923-x, 30043270 PMC6061211

[66] Myśliwiec N Ciesielska A Wojtczak M Sieradzka A Kot A Różycki A . The impact of physical activity on mental health. Qual Sport. (2025) 37:57234. doi: 10.12775/qs.2025.37.57234

[67] Bühler JL Orth U. Rank-order stability of relationship satisfaction: a meta-analysis of longitudinal studies. J Pers Soc Psychol. (2022) 123:1138–65. doi: 10.1037/pspp0000430, 35878099

[68] Dapp LC Orth U. Rank-order stability of domain-specific self-esteem: a meta-analysis. J Pers Soc Psychol. (2024) 127:432–54. doi: 10.1037/pspp0000497, 38451709

[69] Hu C Huang Y Zhang W. Childhood emotional abuse and suicidal ideation in college students: exploring the mediating role of alexithymia and the moderating effect of physical exercise. Front Psych. (2025) 16:1660164. doi: 10.3389/fpsyt.2025.1660164 PMC12698450 41393228

[70] Hu C Bin J Zhang W Huang W. How sports-implied packaging of protein powder products enhances the purchase intention of generation Z: evidence from multiple experiments. Front Nutr. (2025) 12:1645614. doi: 10.3389/fnut.2025.1645614, 41323995 PMC12661654

